# “The more you learn, the more you can influence”—learning circles to support citizen science in Parkinson's disease: a pilot study in Sweden

**DOI:** 10.3389/fpubh.2025.1717528

**Published:** 2026-01-14

**Authors:** Jamie Linnea Luckhaus, Therese Scott Duncan, Carina Hellqvist, Sara Riggare

**Affiliations:** 1 Participatory eHealth and Health Data Research Group, Department of Women's and Children's Health, Uppsala University, Uppsala, Sweden; 2 Research Group for Frail, Critically Ill Patients, Department of Health, Medicine and Caring Sciences, Linköping University, Linköping, Sweden; 3 Department of Neurology, Linköping University Hospital, Linköping, Sweden

**Keywords:** citizen science, learning circles, Parkinson's disease, patient empowerment, personal science, self-care, self-management

## Abstract

**Introduction:**

Parkinson's disease (PD) is the fastest growing neurological condition, making it a public health concern. There is still much to be learned about this complex disease, and citizen science–the involvement of the public in scientific research–has been used for public health initiatives in other conditions. Meaningful engagement in science requires knowledge and skillset to do so, including a foundational understanding of one's condition. Learning circles are a well-established peer-learning format which have been used for patient education in other conditions.

**Aim:**

To explore the potential of learning circles for strengthening self-care and citizen science in Parkinson's disease.

**Methods:**

Four rounds of online learning circles (6–9 persons with PD, 1 h/week over 3 weeks) were held between May–July 2025, with 21 participants completing a whole round, and eight dropped out. Semi-structured interviews were conducted before and after the intervention, and the 16 participants who participated in both were included in the analysis. Data were analyzed using a framework-based longitudinal thematic approach. Participant-by-theme matrices captured individual change and group patterns, and findings were synthesized under the three World Bank's pillars of empowerment (resources, agency, context), with color coding distinguishing timepoints.

**Results:**

Resources: Participants described gaining new knowledge and mindset of PD on a general and personal level, in part through peer-learning. Agency: Participants expressed self-efficacy and began seeing themselves as the main driver of their care, resulting in (re-)engaging in self-care. Context: The dual researcher-PwP role of the facilitator proved crucial. The digital format was appreciated, and challenges in healthcare and society were discussed.

**Conclusions:**

Learning circles show promise as a format for strengthening self-care and citizen science in Parkinson's disease. This participatory approach may advance citizen science by turning lived experience into collective insights.

## 1 Introduction

Parkinson's disease (PD) is the fastest growing neurological condition in the world and is projected to affect over 13 million individuals in 2040 (1), making PD an important public health concern.

Citizen science for health refers to the involvement of the public in scientific research related to health and medicine, and it has been identified as a potential method to improve public health initiatives (2). The degree of involvement on the part of the individual citizens can vary, from low (involvement only in data collection or basic interpretation) to high (participatory approaches where citizens and “professional” researchers share the responsibility and workload of the conducted research). Den Broeder et al. (2) have defined two levels of participatory citizen science approaches: level B; *participatory science*, and level A; *extreme citizen science*. The term *extreme citizen science* describes a model in which non-professional scientists actively shape research questions, collect data, and contribute to analysis and outcomes. This bottom-up approach gives communities agency over scientific processes, especially in areas affecting their lives directly. An extension of this idea is “patient science,” as defined by Heyen et al. (3), where patients play a central role in generating knowledge about their own health conditions. Patient science views patients as experts-by-experience who engage in scientific inquiry to address practical, everyday challenges posed by their illnesses. It overlaps with extreme citizen science in its participatory ethos, but it focuses specifically on health and illness as the domain of inquiry.

However, meaningful engagement in patient science requires a foundational understanding of one's condition. Without such understanding, patients may struggle to formulate relevant questions or interpret data. This is where *personal science* becomes essential (4, 5). Personal science involves individuals conducting systematic self-research using for example self-tracking, experimentation, and data analysis to gain insights into their own health (4).

In this sense, personal science aligns closely with the principles of *self-care*, which emphasize an individual's active role in managing the medical, emotional, and social demands of chronic illness (6). Riegel et al.'s (7) established and recently updated self-care framework conceptualizes this into three key processes: maintenance (healthy routines physically and mentally), monitoring (self-tracking), and management (responding to signs and symptoms). Just as self-care empowers patients to better navigate daily life and treatment decisions, personal science equips them with practical skills and experiential knowledge that can strengthen their participation in—and the overall impact of—collaborative research.

People with PD (PwP) experience a wide range of motor and non-motor symptoms, and treatment regimens become increasingly complicated over time. Treatment may include a variety of therapies, but primarily include oral pharmaceuticals, often consisting of a complicated regimen of multiple different prescriptions and several intakes per day—five or more intakes is not uncommon. Even with personalized and optimized treatment regimens, symptoms and medication effects are often unpredictable, making everyday life with PD challenging. It is therefore essential that PwPs are knowledgeable and active in their treatment decisions and self-care (8–11). Despite growing evidence of the importance of self-care interventions for PwP, self-care remains an under-acknowledged and under-researched field in PD (12), and often focuses on self-care maintenance or management of motor symptoms over a holistic approach (13). However, personal science and citizen science, such as patient-led N-of-1 and self-tracking studies, have gained traction in PD (14–18).

There is still, however, a need to explore structured, community-based formats that can support collective learning and self-care among PwP. Learning circles (LCs) are a well-established peer-learning format for adult education, for example within public health initiatives. LCs, or “study circles” as they have long been called in the Nordic countries (19), are typically organized through non-profit associations and have been applied in patient education, for instance in type 2 diabetes, multiple sclerosis, and mental health (20–23). In the field of mental health, LCs have also been facilitated by people with lived experience as part of an educational intervention in Sweden, inspired by the network of “recovery colleges” in the United Kingdom (23). These programs rest on the core assumption that every individual brings valuable skills, knowledge, and experiences to the group, and that peer learning can foster self-care.

Our hypothesis is that by engaging in LCs—gaining disease-specific knowledge and knowledge of systematic self-research tools—PwP can build both the knowledge base and the confidence needed to engage in personal science and thereby strengthen their abilities to contribute more actively to collective citizen science initiatives. The aim of this study is therefore to explore the potential of LCs for strengthening self-care and citizen science in PD by conducting a pilot study in Sweden.

## 2 Methods

This study employs a longitudinal qualitative approach by comparing interviews pre and post intervention (LCs) and is reported according to the COnsolidated criteria for REporting Qualitative research (COREQ) checklist (24). See Supplementary material for the completed checklist.

### 2.1 Participants

Participants were recruited via PD patient organizations and out-patient clinics in Sweden, where an information text about the LCs and pilot study was disseminated and those who were interested left contact information to receive an email with further sign-up information. Inclusion criteria were: being diagnosed with PD (self-reported), interested in learning more about PD self-care with a focus on medical treatment, and access to a computer or other digital device with internet access. PwP would have been excluded from the study if they were deemed unlikely to be able to participate meaningfully in the LCs, for example due to severe cognitive difficulties, insufficient computer literacy, or insufficient Swedish language skills. No individual who expressed interest was excluded.

### 2.2 Intervention

Four rounds of learning circles were carried out in Swedish over Zoom in a small group format (6–9 PwP per group) over 3 weeks with one online session (1 h) per week. The facilitator was a researcher living with PD with expertise in PD self-care, personal science, and citizen science for health, as well as one of the authors (SR). The first round started May 2025 and the fourth concluded in July 2025. At the start of every session, participants were given the disclaimer that the objective was not to provide medical advice, nor is the facilitator a healthcare professional. The LCs aimed to strengthen participants' ability to make informed care and treatment decisions through brief, informative presentations followed by trust-building, semi-structured discussions. The presentations introduced key topics and sparked conversation, while the discussions allowed participants to explore issues most relevant to them. In each round, there were two homework assignments: (1) (between the first and second session) to read about their PD-related medications on the website collating official information on all prescription medications available in Sweden, and note any of the side effects listed they may have experienced. Participants also read the information intended for healthcare providers (HCPs) on the same website, and noted differences from the patient-facing information. (2) (between the second and third session) participants noted their medication intake and observations related to their physical and mental functioning as they took their medications as well as at least once between medication intakes over the course of a few days using the methods of their choice (pen and paper, Excel, etc.).

### 2.3 Interviews

**Figure 1:**
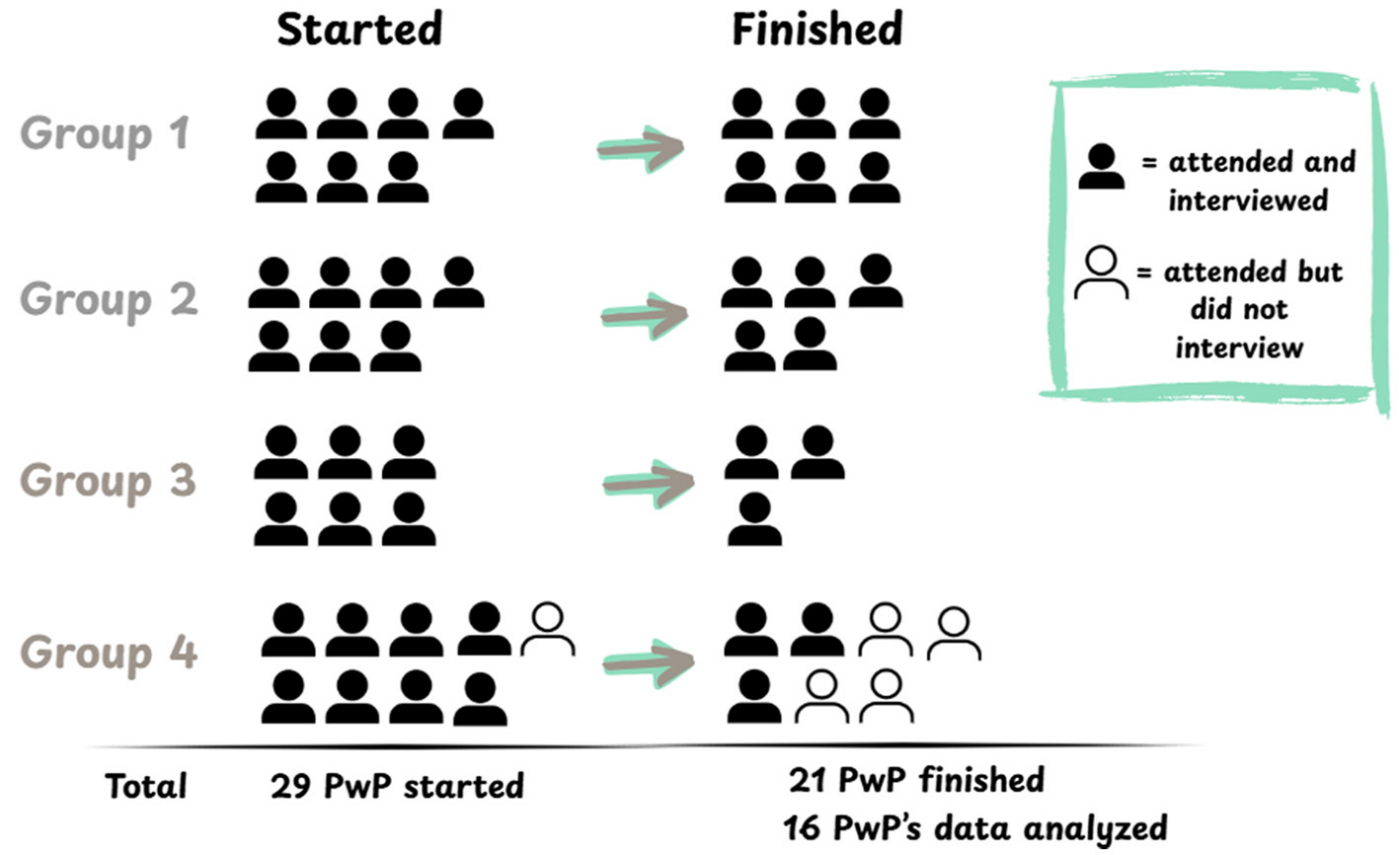
Number of participants in each round from the first to the last meeting. Number who started in each round **(left)**, number who completed the round **(right)** with the solids having conducted a pre- or post-interview, respectively, and the outlined having not re-interviewed.

Semi-structured interviews were conducted pre and post intervention (three sessions of LCs), and analyzed using a framework-based longitudinal thematic analysis (25, 26). A total of 36 individuals responded to the recruitment text and were invited to sign up for a pre-intervention interview with JLL (female PhD candidate, MPH, with qualitative training), and a round of learning circles with SR (female PhD, researcher living with PD). The interviewer did not participate in the intervention, in order to create separation of the intervention and the research for the participants and increase likelihood of honest reflection in the interviews. In some cases, participants knew SR from prior research projects or PD networks, as they are all active members in the PD community. A total of 29 semi-structured pre-intervention interviews were conducted in Swedish over Zoom, and 17 post-intervention (46 total), see Figure 1. One participant completed the LCs without a pre-interview. All 21 participants who completed a round of LCs were asked to interview again after. Only the 16 participants who were interviewed twice were included in the analysis. See Table 1 for characteristics. The average length of the 16 pre-interviews was 21 min (range 9–32 min) and the average of the 16 post-interviews was 29 min (range 8–53 min). See Supplementary material for the interview guides.

**Table 1:** Interview participant characteristics.

**Participants**	***n =* 16**
*M*	2
*F*	14
Age (years)	54–81 (mean 66, median 67)
Years since diagnosis	0–10 (mean 4, median 4)

### 2.4 Analysis

Interviews were analyzed using a framework-based longitudinal thematic analysis, with attention to individual (trajectories) and group-level (recurrent cross-sectional) changes (26). Transcribed using Jojo (27), transcripts were read closely before being coded in *Taguette*^©^. Coding was conducted in Swedish, and where appropriate, *in vivo* codes were used to remain close to the data (28). To ensure comprehensiveness, anything of potential interest was coded under “other” within the framework.

Codes were organized into two side-by-side matrices (pre and post interview) in Miro^©^ (29) structured by interview guide topics (columns) and participants (rows), allowing before and after comparison at an individual level (see Supplementary Table 1). This framework enabled both within-individual trajectories (processes of change) and cross-participant pattern identification (25). Analysis of pre-interviews was completed first and in parallel with the collection of post-interviews, to allow a sense of the group perspectives in isolation.

After discussing individual and group patterns in the matrices and reviewing field notes, the authors agreed that empowerment was a prevalent theme. The data were reorganized, directed into the World Bank's three-pillar framework of empowerment: *resources, agency*, and *context* (30). This framework conceptualizes empowerment as the capacity to make choices and act on them, aligning with our aim of examining how LCs support self-management and citizen science. Pre- and post-interview data were integrated within these pillars, with color coding used to distinguish the timepoints. This integration was chosen to highlight both continuity and change in participants' accounts while avoiding an artificial separation of themes, ensuring temporal context was retained within a broader thematic structure. See an example of the directed thematic analysis process in Supplementary Table 1.

The initial framework analysis allowed us to assess whether individual participants showed evidence of change over time, ensuring that group-level comparisons in the recurrent cross-sectional analysis reflected genuine shifts.

### 2.5 Ethics

This study has been approved by the Swedish Ethical Review Authority (dnr 2025-01066-01). Study information was provided written and verbally, with a chance to pose questions and keep the written information. Informed consent was obtained prior to the first interview.

## 3 Results

The qualitative analysis resulted in nine sub-themes, which are presented under the larger themes: resources, agency and context. Each theme is composed of a combination of pre and post intervention interviews with the participants who completed a full intervention. Translated illustrative quotations are provided.

### 3.1 Resources

Participants were asked to describe their knowledge of PD before and after the learning circles. They described what they hoped to learn and actual learnings, which are presented below. Internal resources, i.e., skills, knowledge and mindsets, as well as external resources, e.g., peers and literature, which impact self-care abilities are described below.

#### 3.1.1 Knowledge of Parkinson's disease

In the pre-interviews, participants described a variety of sources for learning about PD: other PwP, scientific articles, seminars, podcasts, websites, ChatGPT, movies, PD organizations/foundations and support groups, and HCPs. A challenge, however, was finding relevant information on an individual level. Participants noted that there are mountains of information—possible symptoms, causes, progression, treatments, but “which are relevant for me?” They hoped that this question would be answered in the LCs, both in terms of tools for evaluating information sources, as well as concrete, actionable information. When asked to reflect on the LCs, participants described learning about PD in general, that the intervention gave, “*a deeper insight into what it's like to have Parkinson's*” (post-interview 28); but above all, it taught them how to reason about their individual PD. In more general terms, the LCs highlighted the heterogeneity of the disease. Participants reflected how in their group they had different symptoms, treatments, healthcare experiences, and were diverse in regards to age, years with PD, and lifestyle. “*There was such a huge spectrum of individuality*” (post-interview 17). A challenge which emerged from learning more about medication side-effects and tracking symptoms and wellbeing, was that it raised further questions around what is what. Participants asked themselves, are the signs and symptoms due to my PD, my PD medications, or due to aging? Or in the cases of comorbidities, which morbidity is the culprit? The intervention led one participant to reflect over how “*the distinction between the disease and side-effects from the medication was very difficult. It's hard to draw a line. What is what?*” (post-interview 36). Some participants mentioned this challenge already before the intervention, as something they had hoped to learn in the LCs, while others first thought of it upon learning side-effects.

#### 3.1.2 Peers

Another anticipation discussed in the pre-interviews was to meet other PwP. Some participants already had connections with other PwP, while for some this was a less familiar experience. Either way, participants described afterwards that they exchanged practical tips like the best walker model. “*I got some good tips that I have already tested and feel that [PD-related challenge] is no longer a problem”* (post-interview 24). Another participant shared how, until someone in the group mentioned that they swallow their pills dry, this solution had never even occurred to them. Taking pills at work was a great source of stress as the participant's days are unpredictable, making carrying water logistically challenging. The participant was not open about their PD diagnosis, so searching for water can be conspicuous. This seemingly small tip changed the daily life of the PwP. Another learning example from group discussion was when a participant asked the group about a feeling of internal tremors, which the PwP had not found any information on, and thought they were alone in this symptom experience. It turned out, most of the group experienced the same symptom, though also had thought they were the odd ones out. “*That little question still showed how much ignorance there is among us”* (post-interview 9). A newly diagnosed participant felt it was valuable to see how others take in information, since it was often too early to relate personally.

The coping strategy of creating distance from the disease, was transformed for some through meeting the facilitator and other participants. The facilitator, being an employed, socially and professionally active, middle-aged female PwP, was a motivating example for the other participants. Since it is described by one participant that the society's view of PD is crippled old men, participants found it refreshing to meet other PwP that they could relate to.

One participant in particular described that, “*[the intervention] maybe broke some sort of isolation...that life goes on. We got that kind of perspective...Or a better understanding of our own situation (...) not like I've now risen from the dead, but a little like that”* (post-interview 19). The participant began leaving their house again, moving and socializing more. Others described solidarity in others who could empathize with certain symptoms and situations. A participant felt relief in not being alone in the struggle to find motivation to exercise, despite knowing it is one of the few proven actions a PwP can take toward slowing progression.

#### 3.1.3 Mindset toward Parkinson's disease

One's ability to take in information and motivation to seek information to begin with, were described as differing by phases. One participant, who also has Crohn's disease, went through the “*vacuum-journey*” first with Crohn's, and then again with PD, constantly seeking information, almost obsessively. Another participant shared, “*I have a high level of knowledge about PD. In the beginning I read a lot, but now I want to rest and live, and am focused on optimizing my everyday life” (pre-interview 28)*.

Having trust for one's neurologist–another key resource–meant being able to tune out, and spend less energy of one's own on self-tracking, participants explained. The intervention, however, prompted one participant to take a more active role going forward. “*I feel safe with my doctor, so that I've [been able to] try to have fun, something that makes me feel better, rather than delving into these symptoms. What different effects it can have. But I realize that I also have to take... take part in it*.” (post-interview 43). The mindset and other resources participants had at present played a critical role in applying learnings. Maintaining distance was one strategy described for coping with having PD, “*I hope that [the LC] doesn't consume me. That is, that I think even more about Parkinson's. I hope that I continue on the journey I am on - to keep my distance*.” (pre-interview 21).

A resource participants hoped to gain through participation in the LCs was skillsets on how to talk about their PD. As discussed in the theme *context*, there is misinformation and stigma around PD, which results in participants choosing carefully who to disclose their circumstances to. Out of those employed, not everyone had disclosed their PD at work, which also made the regular medication schedules more stressful. Some participants felt that the LCs equipped them with tools to convey the complexity of PD self-care, and the large proportion done on their own. A participant showed their spouse an illustration from the course, saying “*the slide meant quite a lot to me. It shows how much you have to do when living with this disease”* (post-interview 9).

### 3.2 Agency

Participants described knowledge as power, but not without challenges. This section described topics participants touched on that impact their *agency*, or the ability to make informed choices in regard to their PD, and act on them.

#### 3.2.1 Self-efficacy

When asked about motivation for signing up for study groups, participants often responded because “*You can never learn too much*” (pre-interview 14). There was an expressed hope that by knowing more, despite previous efforts to read up, one could gain better control of the disease and its daily impact. “*Part of creating a better everyday life is getting an overview of your own illness*” (post-interview 17). A participant shared that with time they have learned “*that knowledge, I have to have it so that I myself can contribute to feeling as good as possible (...) knowledge is power, quite simply”* (post-interview 43). Knowledge is empowering, but in the case of PD, there is so much which is unknown, even by top experts. Thus, even a well-read PwP is still left with many questions which can feel debilitating:

“So that I understand this is impossible to understand. Because there is no one who can be clear about it because it is not clear. And I would like someone to say, ‘If you do this, you will be able to do this for ten years'... But there is no one who can say that” (post-interview 22).

Although questions about their future remained unanswered, participants reflected after the LCs, however, that they had learned more about their own PD, including methods to ease everyday life. The medications homework gave “*insight that you yourself can influence the medication based on the side effects. So, the more you learn, the more you can influence”* (post-interview 9). In terms of the complex question of what is what, Parkinson's or other? Some participants expressed having obtained methods (self-tracking) to distinguish for themselves, while others felt the intervention raised yet more unanswered questions, depending on the participant's perspective.

It depends on where one is in their journey and the resources one has, as to whether such behaviors empower or deter. One participant shared, already before the intervention, that without having had the need (PD) to pay attention to patterns in their wellbeing, perhaps they may never have, “*It is thanks to receiving this diagnosis that I have gotten to know my body and begun to understand the signals.”* (pre-interview 43). While some feared the course could cause additional burden, others hoped it might bring acceptance. “*I have hope that I will become even more confident in my illness [and] accept that I have the illness” (*pre-interview 43*)*.

Participants described both before and after the course, the burden of knowledge-seeking and active self-management. “*I'm not planning to read anything else. I've closed my books. Now I'll do what the doctors say and what you say in the training”* (pre-interview 32). When asked in the pre-interviews how participants manage their symptoms, one responded “*I take my medication as prescribed. I don't experiment on my own with it.”* (pre*-*interview 4). The degree of freedom felt to alter treatment, whether adjusting the timing, cutting out a dose, taking medications close to a meal, or other, varied between participants. One PwP shared in the post-interview that they had been to their neurologist since the LC, and asked about the concept of a “daily dose,” something that was discussed in the group. To their dissatisfaction, they did not feel they got a clear answer from their neurologist, and therefore could not yet attempt to make any adjustment to their regime. Another participant expressed skepticism, asking where the line should be drawn.

“This idea of being able to control, being able to replenish dopamine, your own dopamine, and in that way, you could correlate it with lowering the dose. Then you're really on slippery ice. It's hard to decide for yourself” (post-interview 36).

A participant who had already self-discovered that taking medications tighter together improved their wellbeing, mentioned that not everyone would dare to make a change. The PwP referred to a neighbor with PD, who did not question when the doctor forgot to refill a prescription and instead went on without it until the participant called on the behalf of the neighbor.

Other participants described these discussions almost as permission to make adjustments and freeing them from the strict and burdensome scheduling they had long followed. “*I used to follow the times slavishly*,” one participant explained, but is now less strict with the medication times. “*So that's what I've changed, that I've become freer in my thinking”* (post-interview 8). Another participant described their most valuable take-away as “*that you can actually control your own illness in a way. Or that you don't need to be so rigid when it comes to these different aspects and maybe question them a little.”* (post-interview 19).

#### 3.2.2 Self-care activities

Self-care strategies participants described before the LCs were vast, with a strong theme of planning. Participants began the first interviews by describing how PD impacts their daily lives, including which symptoms are most burdensome. Although participants face many motor symptoms, it was the less visible symptoms—fatigue “*that you can't sleep away”* and getting easily stressed—that many felt impacted quality of life most. “*The physical part is difficult, but (...) It's the mental part that's the hard part.” (pre-interview 9)*. In terms of motor symptoms, it was poor balance and “*heaviness*” of muscles creating “*the inability to move*” and making hobbies near impossible. As these symptoms are a daily challenge, PD requires planning and routine, navigating medication schedules, energy fluctuation, exercise classes, and more.

“*I have to plan all the time. How I organize my day to have energy and to make sure it goes well. Often, it's like one activity per day*” (pre-interview 9). Simply put, PD “*encompasses all of life*” and impacts “*everything I do - how I sleep and how I am awake*” (pre-interview 9).

Planning meals around medication schedules, planning social and work life around energy levels, being sure to always carry water, and planning for possible future disease progression, planning was a skill participants implemented daily. Participants also mentioned exercise, including with PD groups, as well as meeting specialists such as physiotherapists and speech therapists. Participants were doing everything in their power to feel the best they could and prevent any worsening, “*there's exercise and medicine. If there's any other way, please let me know”* (pre-interview 22). In terms of medication management, some had already experimented and found adjustments that made them feel better, while others felt that was the neurologist's job and none others.

In terms of introspection, participants felt that the LCs gave them new insights about their own PD, through self-tracking and finding new patterns in their symptoms and effects of medications. A participant suffering from dyskinesia (a side effect of PD medications), self-tracked symptoms in relation to medication doses, and decided to discontinue a dosage of levodopa/benserazide. “*I actually did [alter my medication] without talking to a neurologist. That's how I did it. I'm going to visit him in July. (...) So I've discontinued a “quick-dose” that gave me dyskinesia* (post-interview 17). After continuing to monitor, the participant found that the dyskinesia decreased, without any worsening of other symptoms. The PwP plans to show these notes in their next doctor's visit. Other participants shared patterns in stress levels and PD symptoms, food-to-medication timing's effect, and dose timing's effect on energy levels. In summarizing the LCs, participants felt it was different from other educational programs they had taken part in; it provided a new way to think, “*a different angle.”*

#### 3.2.3 Being the expert of your own Parkinson's

Some participants exhibited a realization that they are an expert in their own health. “*This way of integrating patients into the process. It's very good. I'm not a special patient. But I am a patient who has Parkinson's and I have a little bit to say about my way of living with Mr. Parkinson everyday.”* (post-interview 26). A means of structuring and unlocking this expertise about one's PD that was exemplified in the intervention was self-tracking. The aspects discussed above under *resources* helped participants become more active patients and gave the feeling of control.

Part of gaining control was reflecting over one's own role in their care. As a PwP, “*you have to work on it every day, that is, practice self-care every day … It's actually such a small part, [that] the doctor sees me*” (post-interview 14). Participants realized that they are the primary drivers of their care, not HCPs. Although participants varied in their activity level of self-care both before and after the intervention, across the board, participants described an intention to be more active in preparing for and navigating healthcare visits. Having a structured summary of tracked wellbeing, symptoms, and medications to justify any requisitions or changes were viewed as building credibility, and an important addition to the list of questions some already used to prepare for their neurologist. Whether participants planned to track long term or not, it was seen as important to do a week or more prior to a visit in order to better know their own state, and better help their doctor to help them.

“*I have to be prepared because, how in the world could he know? He has like 2,000 patients. That hour is going to be so important. Yeah, unreal, this naivety of mine (...) How much easier it could be for both of us if I'm well prepared. That became very clear to me”* (post-interview 17).

Without self-tracking, i.e. taking notes in some form, one participant explained, “*...you forget about [signs and symptoms]. Your alarm sounds and. (...) then you take [your medicine] and then you continue*” (post-interview 37). Tracking makes the doctor's visit easier for both parties.

### 3.3 Context

The broader context, including organizational structures and culture within healthcare, societal expectations and stigma, and systemic barriers, which impact how participants are able to practice self-care, are described below.

#### 3.3.1 Learning circles format

Although an in-person format was recognized as allowing faster connection-building, participants appreciated the digital format of the LCs. Not only could participants living in more remote areas attend, but remote meetings reduced the risk of cancellation due to fluctuating health, and participants could choose how much of their condition to disclose, rather than being visibly marked by aids such as walkers.

Another valued aspect of the LCs was the ethos of the facilitator, given their dual perspective as a PwP and an academic. The former meant participants could relax and not feel judged nor have to explain symptoms, while the latter meant that there was no concern over misinformation. One participant shared how patient support groups, although they can be socially fulfilling, tend to result in sharing tips and weighing in on topics such as medications which they are not qualified to speak to. That the facilitator “*has the disease themselves, [the educational material] becomes more, it sounds strange, but more credible.”* (post-interview 43).

#### 3.3.2 Parkinson's in healthcare

In relation to healthcare, participants frequently experienced shortcomings. One common concern was the lack of adequate time with healthcare professionals (not only neurologists, but also nurses and other providers); that you come to the clinics twice a year to get your medication and leave. Another was the feeling of only being listened to when it is acute. “*I'm a little skeptical toward healthcare, but of course, if it's something catastrophic, I think they'll listen*” (post-interview 19). Not everyone felt that their concerns were seen, “*Healthcare also seems to have a bit of difficulty understanding that there are symptoms that are not visible*.” (post-interview 9).

Despite the aforementioned challenges, participants described positive experiences of healthcare, particularly regarding having trust and communication with their neurologist. Those who had an interdisciplinary Parkinson's Team—consisting of MDs, nurses, physiotherapists, counselors, speech therapists and occupational therapists who collaborate in providing PD care, with a primarily advisory role—were pleased, and a participant who had recently relocated was excited to get access to a Team.

#### 3.3.3 Parkinson's in society

A continuous source of exhaustion is the stigma and ignorance in society regarding PD.

“*People think you're supposed to be a 75-year-old man standing there shaking with a cane. But that's not how it is... It's a bit tiring to keep explaining that. Because what you encounter is, a common comment is that it's not obvious that I have Parkinson's. That I don't shake like that. That's how people think it should be, but that's not how it is” (post-interview 09)*.

Such misconceptions of how PD presents have also translated into challenges in managing daily life; as one participant shared, who had tried numerous times to get a handicap parking pass, but was told they did not appear disabled enough to need one. Another challenge participants faced was the lack of knowledge regarding non-motor symptoms of PD.

*If you ever try to explain to someone who is not familiar with [PD], you almost end up having to sit and argue that you have cognitive problems. Because you might be in a happy and pleasant context and you are happy and pleasant at that moment, then you go home and faint.” (pre-interview 9)*.

This lack of understanding contributes to many PwP not disclosing their disease, or else carefully choosing who they tell. “*There is still a lot of ignorance and prejudice around the disease. Both within healthcare and in the general public. So, I usually keep quiet about the fact that I have the disease*” (post-interview 36).

## 4 Discussion

This study explored the potential of LCs to support self-care and citizen science in Parkinson's disease. Our findings suggest that LCs have the potential to create a space for participants to exchange experiences and implement concrete strategies related to living with PD. In terms of the three pillars of empowerment, participants not only gained new knowledge (resources), but also confidence to act on it (agency). The LCs enabled reflection and discussion over their condition and care (context) in ways that extend beyond traditional clinical encounters. Our study highlights how LCs may function not only as peer-learning, but as supporting self-care and ultimately citizen science.

### 4.1 Empowerment: translating knowledge into action

Empowerment in chronic illness is often framed as the ability to turn knowledge into practical action (31). Drawing on the World Bank's three pillars of empowerment—*resources, agency*, and *context*—our findings suggest that empowerment is central to bridging personal science and citizen science: while citizen science emphasizes knowledge production, empowerment represents the ability to act upon that knowledge.

Our findings suggest that LCs supported PwP in mobilizing both internal resources (resilience, self-insight, self-care skills) and external resources (networks, professional advice, tools) (12), strengthening their *agency* to face challenges like healthcare limits and stigma (*context*). Participants described gaining new insights into their symptoms and medications, as well as newfound methods and motivation to self-track and structure their individual observations for themselves and their HCPs. This enhanced capacity for systematic self-research, or personal science, equips PwP with the practical skills and experiential knowledge necessary for engagement in collaborative research. By pooling these individual insights, the LC format enables participants to generate collective knowledge that may hold value beyond the group itself, advancing the goal of turning lived experience into collective insights. Such insights may enable participants to maximize the value of their limited time with HCPs, focusing on complex and relevant questions and taking part in shared decision-making. In turn, this could reduce the burden on healthcare systems by decreasing repetitive explanations, unnecessary visits, or poorly targeted interventions. Essentially, empowering patients to act on their own observations and reasoning turns self-care into a partnership with healthcare providers. This realization that participants are the primary drivers of their own care and experts-by-experience is closely aligned with the ethos of patient-centered care and citizen science in chronic disease care (2).

Participants also described different phases of information-seeking, where newly diagnosed go through a “vacuum-journey” of non-stop searching and learning. Some further along were now ready to “close their books” and take a mental break from focusing on PD more than necessary. Still, others felt that continuous learning is a way to maintain a sense of control. These findings echo research on *illness identity*, the degree to which a chronic condition is integrated into a person's self-concept (32), and parallel observations that self-care practices often fluctuate over time (12). Importantly, the LCs encouraged some participants to re-engage in self-care maintenance and monitoring while facilitating self-care management, aligning with Riegel et al.'s framework (7), and suggesting that this format can positively influence both disease management and wellbeing. Studies in other conditions, such as IBS, found that acceptance of a disease as part of one's identity supports overall self-care (32).

Beyond individual benefits, LCs create opportunities for PwP to co-produce knowledge and reflect on their lived experience in structured ways. The realization by participants that they gained “insight that you yourself can influence the medication based on the side effects” is particularly significant, as this empowerment fuels the momentum for greater involvement and collective advocacy, which is key to participatory citizen science. This resonates with public health citizen science projects such as Den Broeder et al. (2017), where participants in a disadvantaged neighborhood reported not only increased health literacy (skills and capacities to exert control over health), but also a stronger ‘sense of coherence', i.e., experiencing life as comprehensible, meaningful, and manageable (33). Similarly, our participants described realizing that they are the primary drivers of their own care. This shift in agency is closely aligned with the ethos of citizen science, though it also depends on access to sufficient resources for such responsibilities to be realistic.

Together, these findings suggest that LCs not only strengthen self-care for PwP but also can enable them to act as co-investigators of their own health and care systems. By pooling individual insights, the LC format enables participants to generate collective knowledge that may hold value beyond the group itself.

#### 4.1.1 Learning circles in practice

Our facilitator's dual role fostered trust and discussion, and she began the LCs with her backstory as a patient, an advocate, and a researcher. Participants were also encouraged to introduce themselves and converse, which was why a small group (6–8) was important. The discussions had the presentations as a point of departure, but were open enough to allow participants to explore topics important to them, which also facilitates trust and openness. A similar format of sharing, information, and semi-structured discussion would likely foster trust and openness with other facilitators.

Future LCs might consider a HCP, which would also enable more medical questions and discussion. However, a HCP present could impact the degree to which participants open up about their healthcare experiences, and this was a commonly discussed challenge. Using a HCP may also risk the format becoming more of a lecture than peer-learning, citizen science, which serves another purpose. Therefore, a HCP with a neurological condition would be ideal, and maintain the *peer* learning element. Another possibility is to involve (former) carers to PwP, as explored by Geerlings et al. (34) for peer-support, and provide additional training, if necessary, in the academic aspects of the facilitator role. A carer, although lacking first-hand *patient* experience, can bring valuable insights into daily life with PD and foster trust in conversations about the healthcare system.

If a “peer” facilitator is unavailable, another possibility is a “flipped-classroom” (35) format, where participants receive materials (e.g., video or readings) as homework, and then deepen this knowledge through guided group work and hands-on activities.

In terms of the digital format, other studies have found similar benefits to online PwP group meetings, though they were primarily focused on the peer-support aspect, as opposed to the learning (36). In our study, participants valued being able to join regardless of location or symptom severity, and the data suggested a de-stigmatizing effect, since it was not apparent, for instance, who used a walker or who required maximum volume to participate. The study therefore highlights the suitability of online LCs for PwP. In contrast, in-person formats may be more suitable for other chronic conditions where symptoms are less visible and travel is less burdensome.

### 4.2 Strengths and limitations

A strength and distinctive feature of our study was the facilitator's dual identity as both a PwP and a researcher. Participants emphasized that this combination was crucial: the academic perspective grounded the discussions in scientific merit, while the lived experiences, perhaps even more importantly, created a safe, credible environment for sharing.

Participants who dropped out expressed that it was due to scheduling conflicts, which is difficult to do anything more about than offering multiple sessions and online.

In regard to citizen science, and our aim to strengthen PwPs' abilities to contribute more actively to collective citizen science, one limitation is that we did not explicitly explore participants' willingness and perceived abilities to contribute more actively to collective citizen science via direct interview questions. However, the observed increases in agency and resources inherently establish the potential and readiness for future engagement.

A consideration in interpreting these findings, is the short duration (1 h × 3 weeks) of the LCs. Although this also makes it more feasible to implement in terms of resource allocation, participants expressed there being too few meetings. Additionally, follow-up (interviews) was carried out days to a couple weeks maximum after the end of the intervention, meaning that in most cases, participants were not given ample time to apply learnings or visit their clinician. However, the interviews suggest that this short amount of time was enough to make a difference in thought processes and actions.

Additionally, hard-to-reach groups may be in greatest need of support yet were likely under-represented in our sample. Language barriers and the use of digital recruitment may have excluded individuals with limited digital literacy or access. A narrative review of digital peer-support interventions found that in many cases PD hindered use of technology, suggesting those individuals were likely unintentionally excluded (36). PwP with less technological access might have valued the LCs differently.

To enhance equity and participation, previous research indicates importance of partnering with community organizations (e.g., churches, cultural associations, clinics) to increase awareness of research opportunities, alongside offering both local and remote delivery options to reduce burdens related to transportation, time, cost, and caregiver needs (37, 38). Such approaches may improve reach among underserved PwP populations.

Although cognitive impairment was an exclusion criterion in this study, future LCs could be adapted through adjustments to pacing and structure (more breaks, no homework), simplified and concrete language, multimodal visual materials, and the involvement of caregivers or trained facilitators (39).

Our sample was predominantly female, reflecting common participation patterns in research. To recruit more men, future LCs could be offered through neurology and primary care services as well as community settings that engage men specifically (e.g., veterans' groups, sports clubs). Notably, several women in this study reported that previous information sessions they had attended in Sweden were geared more toward older men, which may explain the perceived relevance and value of this intervention among female participants.

### 4.3 Clinical implications

These findings carry implications for how LCs and similar participatory formats might be integrated into PD care and research. LCs may serve as a low-cost complement to outpatient care models by providing accessible and relevant support—particularly for underserved populations. By fostering self-tracking and reflections, LCs prepare patients to use clinical time more effectively, focusing visits on problem-solving and shared decision-making rather than repetitive explanations; this aligns with broader goals of patient-centered care and sustainable healthcare. In turn, LCs might reduce unnecessary visits or poorly targeted interventions, easing the burden on healthcare systems.

In terms of LC topics, the study outcomes identify areas for future collective citizen science initiatives. A recurring challenge for participants was distinguishing between PD symptoms, medication side effects, and effects of aging/comorbidities (“What is what?”). LCs could be leveraged as a collective effort of systematically self-tracking specific ambiguous symptoms (like internal tremors) in relation to medication timing and external factors. This pooled, structured data could then be collectively analyzed (extreme citizen science/patient science) to generate new, patient-validated patterns of symptom etiology. Additionally, LCs could enable co-design of patient advocacy initiatives (e.g., creating resources for HCPs or the public based on commonly experiences societal misconceptions) to address challenges described related to context.

#### 4.3.1 PD-specific vs. translatable insights

Some of the insights from our LCs are specific to PD, such as the focus on medication timing, fluctuations, and the interplay of motor and non-motor symptoms. PwP often need to fine-tune the timing of levodopa, plan meals and water access around doses, and track “on/off” fluctuations to plan activities and work around—making medication-management highly complex compared to many chronic conditions. LCs focused on personal science can equip individuals with the tools to optimize such planning, as well as share tips, such as how one participant began swallowing pills dry at work after the LCs, and another discovered a pattern in energy fluctuations through the diary assignment. Additionally, as PD is more common in older adults, PwP are likely to live with comorbidities, as several of our participants did, making personal science and daily life that much more complex.

At the same time, many of the benefits—empowerment, peer support, lifestyle strategies, and the value of dual-role facilitators—echo findings from LCs in other chronic conditions (23). Our results align with the Tailored Self-Management Support (TEDSS) framework, developed in collaboration with persons with neurological conditions, indicating that our study design is likely transferable to other neurological conditions (40). TEDSS emphasizes tailoring self-management support to the individual's stage of disease and personal context, which LCs enable. This suggests that while the content of LCs should be tailored to PD, the underlying mechanisms are broadly translatable to chronic illness management.

## 5 Conclusion

This study explored the potential of LCs to strengthen self-care and citizen science in Parkinson's disease. Our findings indicate that LCs can empower participants through peer-learning, structured reflection, and personal science practices, while the dual role of the facilitator was particularly important in combining credibility with lived experience.

The digital format proved especially suitable for PwP, enabling participation regardless of location or symptom severity. Although aspects such as medication timing and symptom fluctuations are specific to PD, broader mechanisms of empowerment and peer-learning are relevant across chronic conditions. Importantly, LCs created opportunities for participants to co-produce knowledge, transforming individual experiences into collective insights with value beyond the group itself—an approach that reflects the principles of citizen science. By enabling participants to translate knowledge into action and prepare for more meaningful clinical encounters, LCs may improve daily management, support patient–clinician partnerships, and reduce strain on healthcare systems. As one participant concluded: “The more you learn, the more you can influence.”

## Data Availability

The raw data supporting the conclusions of this article will be made available by the authors, without undue reservation.
