# Supplementary material for: “The more you learn, the more you can influence”—learning circles to support citizen science in Parkinson's disease: a pilot study in Sweden
Source: Front Public Health. 2026 Jan 14;13:1717528. doi: 10.3389/fpubh.2025.1717528 (PMC12847296; doi:10.3389/fpubh.2025.1717528)
Supplement: Supplementary file 1 [file Data_Sheet_1.pdf]

COREQ (COnsolidated criteria for REporting Qualitative research) Checklist

A checklist of items that should be included in reports of qualitative research. You must report the page number in your manuscript where you consider each of the items listed in this checklist. If you have not included this information, either revise your manuscript accordingly before submitting or note N/A.

Topic	Item No.	Guide Questions/Description	Reported on Page No.
**Domain 1: Research team and reflexivity**			
Personal characteristics			
Interviewer/facilitator	1	Which author/s conducted the interview or focus group?	4
Credentials	2	What were the researcher's credentials? E.g. PhD, MD	1
Occupation	3	What was their occupation at the time of the study?	4
Gender	4	Was the researcher male or female?	4
Experience and training	5	What experience or training did the researcher have?	4
Relationship with participants			
Relationship established	6	Was a relationship established prior to study commencement?	4
Participant knowledge of the interviewer	7	What did the participants know about the researcher? e.g. personal goals, reasons for doing the research	4
Interviewer characteristics	8	What characteristics were reported about the inter viewer/facilitator? e.g. Bias, assumptions, reasons and interests in the research topic	3
**Domain 2: Study design**			
Theoretical framework			
Methodological orientation and Theory	9	What methodological orientation was stated to underpin the study? e.g. grounded theory, discourse analysis, ethnography, phenomenology, content analysis	4
Participant selection			
Sampling	10	How were participants selected? e.g. purposive, convenience, consecutive, snowball	3
Method of approach	11	How were participants approached? e.g. face-to-face, telephone, mail, email	3
Sample size	12	How many participants were in the study?	4
Non-participation	13	How many people refused to participate or dropped out? Reasons?	4
Setting			
Setting of data collection	14	Where was the data collected? e.g. home, clinic, workplace	4
Presence of non-participants	15	Was anyone else present besides the participants and researchers?	4
Description of sample	16	What are the important characteristics of the sample? e.g. demographic data, date	4, 14
Data collection			
Interview guide	17	Were questions, prompts, guides provided by the authors? Was it pilot tested?	4, supplementa
Repeat interviews	18	Were repeat inter views carried out? If yes, how many?	4
Audio/visual recording	19	Did the research use audio or visual recording to collect the data?	4
Field notes	20	Were field notes made during and/or after the inter view or focus group?	4
Duration	21	What was the duration of the inter views or focus group?	4
Data saturation	22	Was data saturation discussed?	N/A
Transcripts returned	23	Were transcripts returned to participants for comment and/or	4

Topic	Item No.	Guide Questions/Description	Reported on Page No.
		correction?	
**Domain 3: analysis and findings**			
Data analysis			
Number of data coders	24	How many data coders coded the data?	4
Description of the coding tree	25	Did authors provide a description of the coding tree?	4
Derivation of themes	26	Were themes identified in advance or derived from the data?	4
Software	27	What software, if applicable, was used to manage the data?	4
Participant checking	28	Did participants provide feedback on the findings?	N/A
Reporting			
Quotations presented	29	Were participant quotations presented to illustrate the themes/findings? Was each quotation identified? e.g. participant number	5
Data and findings consistent	30	Was there consistency between the data presented and the findings?	5-10
Clarity of major themes	31	Were major themes clearly presented in the findings?	5-10
Clarity of minor themes	32	Is there a description of diverse cases or discussion of minor themes?	5-10

Developed from: Tong A, Sainsbury P, Craig J. Consolidated criteria for reporting qualitative research (COREQ): a 32-item checklist for interviews and focus groups. *International Journal for Quality in Health Care*. 2007. Volume 19, Number 6: pp. 349 – 357

Once you have completed this checklist, please save a copy and upload it as part of your submission. DO NOT include this checklist as part of the main manuscript document. It must be uploaded as a separate file.

Supplementary Material: Interview guides

Supplementary Data 1. Interview guides pre and post (original Swedish)

Intervjuguide: Före patientutbildningen

1. Kan du berätta lite om dig själv?
2. Bakgrund, erfarenheter (arbete och andra), familj mm
3. Kan du berätta lite om din sjukdom och hur den påverkar din vardag?
4. Vilka symtom upplever du som mest besvärande?

Kunskap och uppfattningar om sjukdomen

5. Hur hanterar du din medicinering och symtom i dagsläget?
6. Vilka utmaningar upplever du med att följa din behandling?
7. Hur skulle du beskriva din kunskap om Parkinsons sjukdom och dess behandling?
8. Har du tidigare deltagit i någon utbildning eller informationsträff om Parkinsons sjukdom?

Förväntningar på utbildningen

9. Vad hoppas du få ut av den här patientutbildningen?
10. Finns det något specifikt du vill lära dig mer om?
11. Hur tror du att utbildningen kan påverka din vardag och din sjukdomshantering?

Avslutning

12. Vill du lägga till något?

Intervjuguide: Efter patientutbildningen

1. Berätta för mig om patientutbildningen
2. Hur upplevde du utbildningen?
3. Var den som du hade förväntat dig?
4. Vilka delar av utbildningen tyckte du var <u>mest användbara</u>?
5. Fanns det något du saknade eller som kunde ha <u>förbättrats</u>?
6. Hur har det funkat med formatet? E.g. digitala möten

Förändringar i kunskap och hantering av sjukdomen

7. Hur har din förståelse för Parkinsons sjukdom förändrats efter utbildningen?
   a. På vilket sätt?
8. Har du ändrat hur du hanterar din medicinering och symtom?
   a. Om ja, hur?
9. Upplever du att utbildningen har påverkat din livskvalitet?

Framtida behov och stöd

10. Finns det något du fortfarande känner att du behöver mer information eller stöd kring?
11. Hur tror du att vården kan stödja dig bättre i din sjukdomshantering?
12. Skulle du rekommendera den här typen av utbildning till andra med Parkinsons sjukdom?
    a. Varför/varför inte?

Avslutning:

13. Vill du lägga till något?

Supplementary Data 2. Interview guides pre and post (translated to English)

Interview guide: Before the LC

1. Tell me a little about yourself.
2. Background, experiences (work and others), family, etc.
3. Can you tell me about your PD and how it affects your everyday life?
4. Which symptoms do you find most bothersome?

Knowledge and perceptions about the illness

5. How do you currently manage your medication and symptoms?
6. What challenges do you experience in following your treatment (if any)?
7. How would you describe your knowledge of Parkinson's disease and its treatment?
8. Have you previously participated in any training or information meeting about Parkinson's disease?

Expectations for the training

9. What do you hope to get out of this LC?
10. Is there anything specific you would like to learn more about?
11. How do you think the training could affect your everyday life and your disease management?

In closing

12. Is there anything you would like to add?

Interview Guide: After the LC

1. Tell me about the LC.
2. How did you experience the LC?
3. Was it what you expected?
4. Which parts of the LC did you find most useful?
5. Was there anything you missed or that could have been improved?
6. How did the format work? E.g. digital meetings

Changes in knowledge and management of the disease

7. How has your understanding of Parkinson's disease changed after the LC?
    a. In what way?
8. Have you changed how you manage your medication and symptoms?
    a. If yes, how?
9. Do you feel that the education has affected your quality of life?

Future needs and support

10. Is there anything you still feel you need more information or support on?
11. How do you think healthcare can support you better in managing your disease?
12. Would you recommend this type of education to others with Parkinson's disease?
    a. Why/why not?

In closing:

13. Is there anything else you would like to add?

Supplementary Table 1. Examples of individual change within directed thematic analysis process

INT	Pre-intervention (Sv/**En**)	Corresponding code (Sv/**En**)	Post-intervention (Sv/**En**)	Corresponding code (Sv/**En**)	Sub-theme	Theme
08	*"det är så mycket [information on PD] och det är så komplext så det är svårt. Det är också svårt att lära sig."* ***"There's so much [information on PD] and it's so complex so it's difficult. It's also hard to learn."***	svårt att lära sig om PD då så mycket & komplext **hard to learn about PD as so much & complex**	"Och jag uppfattade det som att det var väldigt olika. Ja, hur mycket mediciner, hur länge man har haft diagnosen. Ja, det var väldigt mycket som var olika så. Det visste jag väl i och för sig. Men det blev bekräftat när man sätter ihop en grupp så verkligen." **"And I understood it to be very different. Yes, how much medication, how long you've had the diagnosis. Yes, there were a lot of things that were different. I knew that in and of itself. But it was confirmed when you put a group together so really."**	LC har bekräftat hur olika PD är för olika individer (diagnos, meds osv) **LC has confirmed how different PD is for different individuals (diagnosis, meds, etc.)**	Knowledge of Parkinson's Disease	Resources
09	*"Jag vill ju både träffa andra [med PD] och lära mig mer, men jag vill ju inte ha Parkinson ... ha med den att göra överhuvudtaget"* ***"I want to meet others [with PD] and learn more, but I don't want Parkinson's... to***	"Jag vill ju både träffa andra [med PD] och lära mig mer, men jag vill ju inte ha Parkinson ... ha med den att göra överhuvudtaget" (in vivo)	*"i vår lilla grupp, då var det en man som frågade om någon annan hade... Man kan ju ha så att det darrar på insidan. Det syns inte, men man känner själv att man darrar på insidan. Och det verkar som att han trodde att han var helt ensam om det som symtomet. Och*	det fanns en symtom som någon i vår grupp trodda han var ensam i, men visade sig att vi alla har fast det finns ingen info om symtomen när man läser standardtexter. **There was a symptom that someone in our group thought**	Peers	Resources

	have anything to do with it at all"<br><br>*"Om man någon gång ska försöka förklara för någon som inte är insatt. Så blir det nästan att man får sitta och argumentera för att man har... kognitiva problem"*<br>***"If you ever try to explain something to someone who is not familiar with it, it almost ends up being like you have to sit and argue that you have... cognitive problems."***	**"I want to meet others [with PD] and learn more, but I don't want Parkinson's... to have anything to do with it at all"** (in vivo)<br>många förstår inte att PD också är icke motoriska symptom.<br>**many people don't understand that PD also has non-motor symptoms.**	*det visade sig att vi alla tre i gruppen hade samma sak. Kände igen det. Han var inte unik, utan han var ganska normal. Och det är ett symtom som jag aldrig har stött på i någon av alla de här standardtexterna"*<br>***"In our little group, there was a man who asked if anyone else had... You can have a tremor inside. It's not visible, but you can feel it yourself that you're trembling inside. And it seems like he thought he was completely alone in it like anyone else. And it turned out that all three of us in the group had the same thing. Recognized it. He's never been normal. of all these standard texts"***	**he was alone in experiencing, but it turned out that we all have it, although there is no information about the symptoms when you read standard texts**		
08	*"man skulle också behöva hjälp med att tänka hur ska jag presentera [PD för andra]."*<br>***"I'd also need help thinking about how I should present [PD to others]."***<br><br>*"det är ganska lätt att prata med andra som har Parkinson (...) Men med de som inte har det eller inte har kommit i kontakt med det. Och inte vet om så*	hur ska jag presentera PD för andra?<br>**How should I present PD to others?**<br><br>Andra relaterar och förstår inte hur det är att ha PD, och ser kanske inte att jag har.<br>**Others can't relate or understand what it's like to have PD, and may not see that I have**	*"Jag skrev det här [i mina anteckningar] att hitta sin egen beskrivning av sjukdomen. Och det tänker jag att det är ju bra om man kan göra det för det är ju svårt när man möter någon att berätta, för det är så komplext. Det går inte att säga något enkelt, utan det är så mycket som hänger ihop. Så då använder hon det här att man får hitta sin egen beskrivning på sjukdomen. Och det tänker jag att det är nog bra att tänka så."* ***wrote [in my notes about] this finding your***	[Facilitatoren] berättade att [det] kan vara bra att "hitta sin egen beskrivning av sjukdomen" och det tycker jag är bra. kan vara svårt att förklara för andra då det är så komplext.<br>**[The facilitator] said that [it] can be good to "find your own description of the disease" and I think that's good. It can be difficult to explain to others because it's so complex.**	Mindset towards PD	Reources

	mycket. Då kan jag uppleva att det är ganska svårt. För det är ju så komplext och så diffust. Och så ser de kanske inte på mig då att jag har det."<br>***"It's quite easy to talk to others who have Parkinson's (...) But with those who don't have it or haven't come into contact with it. And don't know much about it. Then I can find it quite difficult. Because it's so complex and so diffuse. And then they might not see me as having it." (INT 08)***		***own description of the disease. And I think it's good if you can do that because it's difficult when you meet someone to tell them, because it's so complex. You can't say something simple, there's so much that's connected. So then she uses this that you get to find your own description of the disease. And I think it's probably good to think that way."***	har inte hittat min beskrivning ännu men man tänker på det mer och mer varje gång man pratar med någon. svårt att uttrycka mig och för andra att förstå<br>**haven't found my description yet but you think about it more and more every time you talk to someone. difficult to express myself and for others to understand**		
19	*"om du talar om mina förväntningar, så tänkte jag mig att alla små råd om praxis och sånt är välkomna … Trots att man tror att man vet allt möjligt, så gör man ju inte det. Det är inte så enkelt."*<br>***"If you're talking about my expectations, I thought that any little advice on practice and such would be welcome... Even though you think you know everything possible, you don't. It's not that simple."***	alla "små råd om praxis" är välkomna<br>**any "little practical advice" is welcome**	"*… Att man faktiskt kan behärska sin egen sjukdom på något sätt. Eller att man inte behöver vara så rigid när det gäller just de här olika momenten och kanske lite ifrågasätta"*<br>***"… That you can actually control your own illness in some way. Or that you don't have to be so rigid when it comes to these different aspects and maybe question them a little"***	mest värdefullt var förståelsen "*Att man faktiskt kan behärska sin egen sjukdom på något sätt. Eller att man inte behöver vara så rigid när det gäller just de här olika momenten och kanske lite ifrågasätta"*<br>**most valuable was the understanding "That you can actually control your own illness in some way. Or that you don't have to be so rigid when it comes to these different aspects and maybe question it a little"**	Self-efficacy	Agency

17	*”Det är en väldig utmaning. Alltså försöka att kartlägga vad som pågår. … Det är svårt för det är så himla olika. Det tycker jag. Ja. Och i dialog med neurologen också. Det att kunna beskriva hur det är, det är också en utmaning. För att symtomen på för lite och för mycket är så likt. Så det är mycket som provar fel, har jag upplevt."* ***"It's a huge challenge. So trying to map out what's going on. ... It's difficult because it's so different. I think so. Yes. And in dialogue with the neurologist too. Being able to describe what it's like, that's also a challenge. Because the symptoms of too little and too much are so similar. So there's a lot of trial and error, I've experienced."***	svårt att beskriva symptom för neurologen, många symptom är så lik **difficult to describe symptoms to the neurologist, many symptoms are so similar**<br><br>vad är symptom och vad är biverkningar? går ifrån neurologen utan att förstå **What are the symptoms and what are the side effects? I leave the neurologist without understanding.**	“jag har faktiskt [ändrat min medicinering] utan att jag har pratat med någon neurolog. Så har jag gjort det. Jag ska ta honom i juli. Så jag har tagit bort en sån kvick medicin som gjorde mig överrörlig.” ***“I actually did [alter my medication] without talking to a neurologist. That's how I did it. I'm going to visit him in July. (...) So I've discontinued a “quick-dose” that gave me dyskinesia***	Efter utbildningen fortsatt jag att tracka o insåg att en kvick madopark gör mig mer överrörlig så jag tog bort den. mår bättre. jag ska till neuron snart ändå **After the training I continued to track and realized that a quick madopark makes me more hyperactive so I removed it. feeling better. Going to the neurologist soon anyway**	Self-care activities	Agency
INT24	*”[Jag vill få ur utbildningen] mer kunskap faktiskt, också kunskap [om] hur jag ska kunna hantera vardagen på ett bra sätt. Genom andras input. Få lite hopp kanske.*	Jag vill få ut mer kunskap och "hur jag ska kunna hantera vardagen på ett bra sätt genom andras input. Få lite hopp kanske Kring forskning	*”det är ju intressant med en sån här utbildning som leds av en Parkinson-patient själv. Och som då har en rätt så bred kunskap, inte bara om sin egen Parkinson, utan Parkinson som helhet. Hon vet också hur det är att vara patient”*	viktigt att facilitatorn har dubbelperspektiv **important that the facilitator has that double perspective**	Learning Circles Format	Context

	Kring forskning och hur framtida livet kan se ut.” ***"[I want to get out of the LC] more knowledge actually, also knowledge [about] how to handle everyday life in a good way. Through input from others. Get a little hope maybe. Around research and what future life might look like."***	och hur framtida livet kan se ut." **I want to gain more knowledge and "how to handle everyday life in a good way through the input of others. Maybe get some hope around research and what future life might look like."**	***“It's interesting to have a course like this led by a Parkinson's patient herself. And who then has a fairly broad knowledge, not just about her own Parkinson's, but Parkinson's as a whole. She also knows what it's like to be a patient.”***			
43	*”det var den läkaren där som frågade mig vad jag menade när jag sa så att jag tar utmattningen, fokuserar på utmattningen nu, för den kommer jag ur. Och Parkinson tar jag sen om det nu är det. Och då tyckte jag han var lite korkad den här läkaren ... Och då sa han, ’ja men du [namn], hur tänker du, för du har bara en kropp och du har två diagnoser.’ Och då insåg jag ju liksom att nu måste jag tänka om här.”* ***"That doctor there asked me what I meant when I said that I'm taking the fatigue, focusing on the fatigue now, because I'll get over it. And I'll take the Parkinson's later***	när jag fick diagnosen var jag också utmattad. tänkte fokusera på det först och PD sen, för det kommer jag ur iallafall. Läkare sa till mig att jag har bara en kropp o 2 diagnoser, måste fokusera på båda. Lärde mig att lyssna på kroppen **when I got the diagnosis, I was also burnt out. I thought I would focus on that first and PD later, because I'll get through it anyway. Doctors told me I only have one body and 2 diagnoses, I have to**	*”jag också har fått hjälp [av utbildningen] med lite, det här måste ju jag kolla upp. Och varför har inte jag fått [den här informationen om mina mediciner]? För att [biverkningarna] ser tydligen lite olika ut. Men jag inser ju att det är bra att veta vad man stoppar i sig också. Inte bara vad det är för. Men jag har ju haft en bra dialog med min läkare, så jag har inte varit orolig på det sättet, utan det är bara mitt tankesätt. Så för mig gav det väldigt mycket att få den här, vad ska jag säga, nya kunskapen.”* ***“[the LC] gave some help with this, I have to look into this. And why haven't I gotten [this information about my medications]? Because [the side effects] apparently look a little different. But I realize that it's good to know what you're putting in***	Jag har bra dialog med läkaren men bra nya kunskap, att veta vad man stoppar i sig **I have good dialogue with the doctor but good new knowledge, knowing what med you’re consuming**	PD in HC	Context

	if that's it. And then I thought he was a bit stupid, this doctor... And then he said, 'yes, but you [name], what do you think, because you only have one body and you have two diagnoses.' And then I kind of realized that now I have to re-think."	**focus on both. Taught me to listen to my body**	***your body too. Not just what it's for. But I've had a good dialogue with my doctor, so I haven't been worried in that way, it's just my way of thinking. So for me, it was very helpful to get this, what should I say, new knowledge."***			
43	"Jag gick med i förbundet ganska så snabbt … Hade en lokal förening som jag också träffade."<br>**"I joined the union pretty quickly… I had a local association that I also met."**	är väldigt engagerad i förbundet och lokalföreningen.<br>**är väldigt engagerad i förbundet och lokalföreningen.**	*"Vården behöver ju ha den senaste kunskapen så att man är uppdaterad. Det finns väl fortfarande en hel del okunskap och fördomar kring sjukdomen. Både inom vården och i allmänheten. Så jag... oftast tyst om att jag har sjukdomen."*<br>***"Healthcare needs to have the latest knowledge so that people are up to date. There is still a lot of ignorance and prejudice around the disease. Both within healthcare and in the general public. So I... usually keep quiet about the fact that I have the disease."***	Finns fördomar och okunskap kring PD inom vården och allmänheten<br>**Prejudice and ignorance about PD within HC and society**	PD in society	Context
